# [^18^F] Al -NOTA-FAPI-46 and [^18^F] FDG PET/CT in detecting postoperative residual and metastatic lesions of papillary thyroid cancer: a head-to-head comparative study

**DOI:** 10.1186/s13550-026-01494-w

**Published:** 2026-08-06

**Authors:** Taisong Wang, Rui Zhang, Na Sun, Lijuan Shen, Wenli Qiao, Qingqing Huang, Yan Xing

**Affiliations:** 1 Department of Nuclear Medicine, Shanghai General Hospital, Shanghai Jiao Tong University School of Medicine, Shanghai, 200080 China; 2 Shanghai Key Laboratory of Molecular Imaging, Shanghai University of Medicine and Health Sciences, Shanghai, 201318 China

**Keywords:** [^18^F] FAPI-46, [^18^F] FDG, PET/CT, Papillary thyroid cancer, Postoperative residual lesion, Lymph node metastasis

## Abstract

**Background:**

Postoperative staging and residual/metastatic lesion detection of papillary thyroid cancer (PTC) before radioiodine (RAI) ablation remains a critical clinical demand, especially for patients with intermediate to high-risk pathological features. Although 2-[^18^F] fluoro-2-deoxy-D-glucose ([^18^F] FDG) positron emission tomography/computed tomography (PET/CT) is widely applied in thyroid cancer imaging, its diagnostic sensitivity is suboptimal for small-volume, low-metabolic metastatic or residual lesions. Fibroblast activation protein (FAP), specifically overexpressed in tumor stromal cancer-associated fibroblasts (CAFs), has become a novel and reliable imaging target for oncological detection. This study aimed to head-to-head compare the diagnostic efficacy of [^18^F]AL-NOTA-FAPI-46 ([^18^F] FAPI-46) and [¹⁸F] FDG PET/CT in identifying postoperative residual and lymph node metastatic lesions in PTC patients prior to adjuvant ¹³¹I therapy.

**Methods:**

A total of 18 consecutive postoperative PTC patients scheduled for ¹³¹I ablation were prospectively enrolled. All patients underwent paired [^18^F] FAPI-46 and [^18^F] FDG PET/CT within a 1-week interval before treatment. A composite reference standard integrating histopathological results, standardized imaging follow-up (median follow-up duration: 8 months, range: 6–12 months) and continuous clinical serological monitoring was adopted to verify lesion properties. Two experienced nuclear medicine physicians independently and blindly interpreted all PET/CT images in random order, with consensus arbitration for discrepant results. Quantitative imaging parameters including SUVmax and SUVpeak were calculated for all target lesions.

**Results:**

For lymph node metastatic lesion detection, [^18^F] FAPI-46 PET/CT exhibited significantly higher sensitivity (82.3% vs. 29.4%, exact two-tailed *P* = 0.022) and accuracy (78.3% vs. 30.4%) than [^18^F] FDG PET/CT, with fewer false-positive cases (2 vs. 4). The true-positive lymph nodes showed significantly higher SUVmax (4.2 ± 3.2 vs. 1.6 ± 2.6, *P* = 0.015), SUVpeak (3.0 ± 2.1 vs. 1.0 ± 1.7, *P* = 0.005) on [^18^F] FAPI-46 imaging than [^18^F] FDG. Three false-positive bone lesions with increased [^18^F] FAPI-46 uptake was found in two patients. These lesions were confirmed as benign fibroproliferative lesions via CT morphological features and follow-up imaging, and no true bone metastasis was detected in the entire cohort. Patients with confirmed lymph node metastasis had significantly higher serum stimulated thyroglobulin (sTg) levels (79.1 ± 51.1 ng/mL vs. 33.9 ± 36.5 ng/mL, *P* = 0.044). The optimal sTg diagnostic threshold for metastatic lesion screening was 25.9 ng/mL (AUC = 0.862), while the predictive value of this cutoff needs further large-sample validation. All enrolled patients had negative anti-thyroglobulin antibody (TgAb) status(< 15ng/mL) to eliminate serological detection interference. [^18^F] FAPI-46 PET/CT successfully identified occult lymph node metastases in patients with elevated sTg and corrected the false-positive findings of [¹⁸F]FDG PET/CT.

**Conclusions:**

In this prospective pilot study, [^18^F] FAPI-46 PET/CT demonstrated superior diagnostic performance over [^18^F] FDG PET/CT for detecting postoperative lymph node metastatic lesions in PTC patients before RAI ablation, with better capability to distinguish malignant lesions from reactive lymph nodes.

## Background

Papillary thyroid cancer (PTC) is the most prevalent endocrine malignancy worldwide, with a continuously rising incidence in recent decades [1]. Most PTC patients achieve favorable long-term prognosis after total thyroidectomy and subsequent adjuvant radioiodine therapy [2]. However, accurate preoperative ablation staging and early detection of postoperative residual tumor or lymph node metastasis are essential for guiding individualized ¹³¹I treatment strategies and improving long-term disease control, especially for intermediate and high-risk PTC cases with aggressive pathological features.

Conventional postoperative monitoring modalities, including neck ultrasound, plain computed tomography (CT) and diagnostic radioiodine whole-body scan (Rx-WBS), often fail to localize small-volume residual or metastatic lesions, resulting in clinical monitoring dilemmas [3, 4]. According to the 2025 American Thyroid Association (ATA) guidelines [5], [^18^F] FDG PET/CT is recommended for patients with suspected tumor residual or metastasis when radioiodine imaging yields negative results. Nevertheless, [^18^F] FDG PET/CT has inherent diagnostic limitations: well-differentiated PTC lesions usually exhibit low glucose metabolic activity, and small metastatic lymph nodes often present insignificant FDG uptake, leading to false-negative results with a reported false-negative rate of 8%–21.1% [6]. In addition, postoperative inflammatory reactions and lymph node reactive hyperplasia frequently cause non-specific FDG uptake, resulting in false-positive diagnoses and interfering with clinical decision-making. Therefore, more accurate and specific imaging techniques are urgently needed for postoperative PTC staging and lesion detection before RAI ablation.

Fibroblast activation protein (FAP) is a type II transmembrane serine protease highly and specifically expressed on CAFs in the tumor stroma of epithelial malignancies, while it is barely expressed in normal adult tissues. FAP-specific inhibitors (FAPIs) have become novel targeted imaging tracers for tumor stroma imaging. Existing studies have confirmed that gallium-68 (^68^Ga)-labeled FAPI tracers possess higher tumor-to-background ratios than [^18^F] FDG, showing excellent performance in detecting lymph node, pleural and bone metastases in differentiated thyroid cancer [7, 8].

Compared with ^68^Ga-labeled tracers restricted by short half-life and centralized production limitations, half-life of fluorine-18 (^18^F) is 109.7 min, which supports centralized large-scale production, long-distance transportation and delayed imaging, with higher imaging resolution and better clinical practicability. Although [^18^F] FAPI-04 has been preliminarily applied in recurrent differentiated thyroid cancer imaging, prospective head-to-head comparative studies between [^18^F] FAPI-46 and [^18^F] FDG PET/CT for postoperative PTC residual and metastatic lesion detection before RAI ablation are still lacking.

This prospective single-center study aimed to systematically compare the diagnostic efficacy of [^18^F] FAPI-46 and [^18^F] FDG PET/CT in identifying postoperative residual and lymph node metastatic lesions in PTC patients prior to ¹³¹I ablation based on a composite reference standard combining histopathology, long-term imaging follow-up and clinical serological monitoring. The results are expected to optimize preoperative ablation imaging strategies for PTC and provide preliminary evidence for the clinical promotion of [^18^F] FAPI-46 PET/CT.

## Methods

### Study design and patient enrollment

This prospective observational study was approved by the Clinical Research Ethics Committee of Shanghai General Hospital, Shanghai Jiao Tong University School of Medicine (Ethics No.: 2021 − 113) and strictly complied with the 1964 Helsinki Declaration and its subsequent amendments. All enrolled patients provided written informed consent before participation. A total of 18 consecutive PTC patients who underwent total thyroidectomy and were scheduled for adjuvant ¹³¹I ablation therapy between September 2022 and June 2023 were enrolled, all with intermediate or high-risk pathological features indicating potential tumor aggressiveness.

#### Inclusion criteria

(1) post-total thyroidectomy interval ≥ 1 month; (2) Completed 2-week low-iodine diet and 3-week levothyroxine withdrawal, with serum thyroid-stimulating hormone (TSH) ≥ 30 mIU/L before examination; (3) Completed paired [^18^F] FDG and [^18^F] FAPI-46 PET/CT examinations within 1 week before ^131^I ablation; (4) Complete clinical and serological follow-up data.

#### Exclusion criteria

(1) Inability or refusal to sign informed consent; (2) History of other malignant tumors or chronic systemic inflammatory diseases; (3) Pregnancy or lactation; (4) Incomplete imaging or follow-up data.

### PET/CT imaging protocols and reconstruction parameters

[^18^F] FAPI-46 tracers were independently synthesized in the Nuclear Medicine Department of Shanghai General Hospital, and [^18^F] FDG tracers were purchased from Shanghai Atomsun Pharmaceutical Co., Ltd. All patients completed two PET/CT examinations with an interval of no less than 24 h to avoid tracer residual interference. Imaging was performed using a uMI780 PET/CT system (United Imaging Medical, China).

For [^18^F] FDG PET/CT examination, Patients were required to fast for 6 h before tracer administration, and blood glucose was controlled between 3.9 and 6.1 mmol/L. No special preoperative preparation was required for [^18^F] FAPI-46 PET/CT. The intravenous injection dose of both tracers was 3.7 MBq/kg. Whole-body PET/CT scanning was performed 60 min after tracer injection.

CT acquisition parameters: tube voltage 120 kV, tube current 250 mAs, layer thickness 3.00 mm, layer spacing 5 mm, pitch 0.813, conventional dose scanning mode.

#### PET acquisition and reconstruction parameters

whole-body 3D acquisition mode, single-bed scanning time 2 min. Ordered subset expectation maximization (OSEM) reconstruction algorithm was adopted, with 4 iterations and 16 subsets. Gaussian post-reconstruction smoothing with a full width at half maximum (FWHM) of 5 mm was applied, and the matrix size was 256 × 256. All reconstruction parameters were consistent for all patients and two tracers to ensure the accuracy and comparability of SUV values.

### Image interpretation and lesion segmentation

Two nuclear medicine physicians with 5 years and 10 years of clinical imaging experience independently completed double-blind interpretation of all images. The interpreters were blinded to all clinical information, including patient age, gender, pathological stage, sTg level, TgAb status and other imaging results. All [^18^F] FDG and [^18^F] FAPI-46 images were randomly sorted before interpretation to eliminate sequence bias. For inconsistent interpretation results between the two physicians, a senior nuclear medicine physician with 15 years of experience participated in discussion to reach a unified consensus diagnosis.

All suspected focal abnormal tracer uptake lesions were included in the analysis. Lesion segmentation was performed based on fused PET/CT images, taking CT morphological abnormal areas as the anatomical reference. A spherical volume of interest (VOI) was manually delineated covering the entire lesion range to avoid inclusion of normal surrounding tissues. Semi-quantitative parameters including SUVmax, SUVpeak of each lesion were automatically calculated by the supporting system software. Lesions with SUVmax below the background of mediastinal blood pool were marked as not applicable (NA).

### Composite reference standard

Considering the limitations of clinical invasive biopsy (small lesion size, deep anatomical location and high biopsy risk), a standardized composite reference standard was adopted for lesion efficacy evaluation, with a clear follow-up cycle definition. The median imaging and clinical follow-up duration of all enrolled patients was 8 months, with a range of 6–12 months. The reference standard was divided into three priority levels:

1. Histopathological confirmation (highest priority): A total of 6 suspected lymph node lesions were confirmed by postoperative puncture or surgical biopsy pathology, which was the gold standard for malignant lesion judgment.
2. Long-term imaging follow-up confirmation: For the remaining 17 unbiopsied suspected lesions, dynamic monitoring was performed via high-resolution neck ultrasound, Rx-WBS, CT or MRI. Lesions with progressive enlargement/increased number during follow-up or significant regression after anti-tumor treatment were defined as malignant; lesions with stable size or spontaneous regression without anti-tumor treatment were defined as benign.
3. Clinical serological auxiliary verification: Dynamic changes of postoperative sTg and TgAb levels were combined to assist in verifying lesion properties, and all patients in this study had negative TgAb status to eliminate serological detection interference.

### Statistical analysis

SPSS 25.0 statistical software was used for all data analysis. Continuous variables conforming to normal distribution were expressed as mean ± standard deviation (SD), and non-normal distribution variables were expressed as median (interquartile range, IQR). Categorical variables were summarized by frequency and percentage. Independent-sample t-test was used to compare sTg levels between metastatic and non-metastatic groups. Paired t-test was applied to compare SUVmax and SUVpeak of true-positive lymph nodes between the two tracers. McNemar’s test(exact two-tailed P-value) was used for paired lesion-level data to compare the diagnostic sensitivity, specificity and accuracy of the two imaging methods, adapting to the small sample size of this study. Receiver operating characteristic (ROC) curve analysis was performed to evaluate the diagnostic efficacy of sTg and [^18^F] FAPI-46 SUV parameters for metastatic lesions. The optimal diagnostic cutoff value was determined by maximizing the Youden index, and 95% confidence intervals (CIs) of AUC were calculated. A two-tailed *P* < 0.05 was considered statistically significant.

## Results

### Patient characteristics

A total of 18 PTC postoperative patients were enrolled in this study, with detailed baseline clinical, serological and imaging data shown in Table 1. All patients had negative TgAb status. The abbreviations in the table are uniformly defined as: pTN (pathological tumor-node stage), sTg (stimulated thyroglobulin), FDG ([^18^F] FDG PET/CT imaging result), FAPI ([^18^F] FAPI-46 PET/CT imaging result).

**Table 1:** Characteristics of 18 patients

No	Age	Sex	pTN Stage	sTg (ng/mL)	TGAb (IU/mL)	Risk stratification	FDG	FAPI
1	37	Male	pT1bN1b	45.7	13.6	3	-	+
2	43	Female	pT1bN1a	121	11.6	3	+	+
3	32	Male	pT2N1b	167	13.1	3	-	+
4	29	Female	pT1aN1a	33.2	12.0	3	-	-
5	31	Female	pT3bN1b	55.9	12.2	3	+	-
6	44	Female	pT1aN1a	10.6	10.9	2	-	-
7	34	Female	pT1bN-	12.7	12.8	2	-	-
8	21	Female	pT1bN1b	23.5	12.2	2	+	-
9	41	Male	pT4aN1a	26.7	10.9	2	+	+
10	46	Male	pT1aN1a	59.9	12.2	3	-	+
11	37	Male	pT1aN1b	123	12.0	3	-	+
12	31	Male	pT1bN1b	134	12.4	3	+	+
13	44	Female	pT1aN1b	11.7	13.6	2	-	-
14	28	Female	pT1aN1a	20.6	11.3	2	-	-
15	38	Female	pT2N1a	24.8	14.8	2	-	-
16	61	Female	pT4aN-	25.2	11.6	2	-	-
17	31	Male	pT1bN1b	34.9	12.0	2	+	-
18	37	Female	pT1bN1a	41.4	12.9	2	-	-

risk stratification = 2 indicates low-risk, risk stratification = 3 indicates intermediate-high risk; “+” indicates positive imaging findings, “-” indicates negative imaging findings

### Diagnostic efficacy for lymph node lesions

**Fig. 1:**
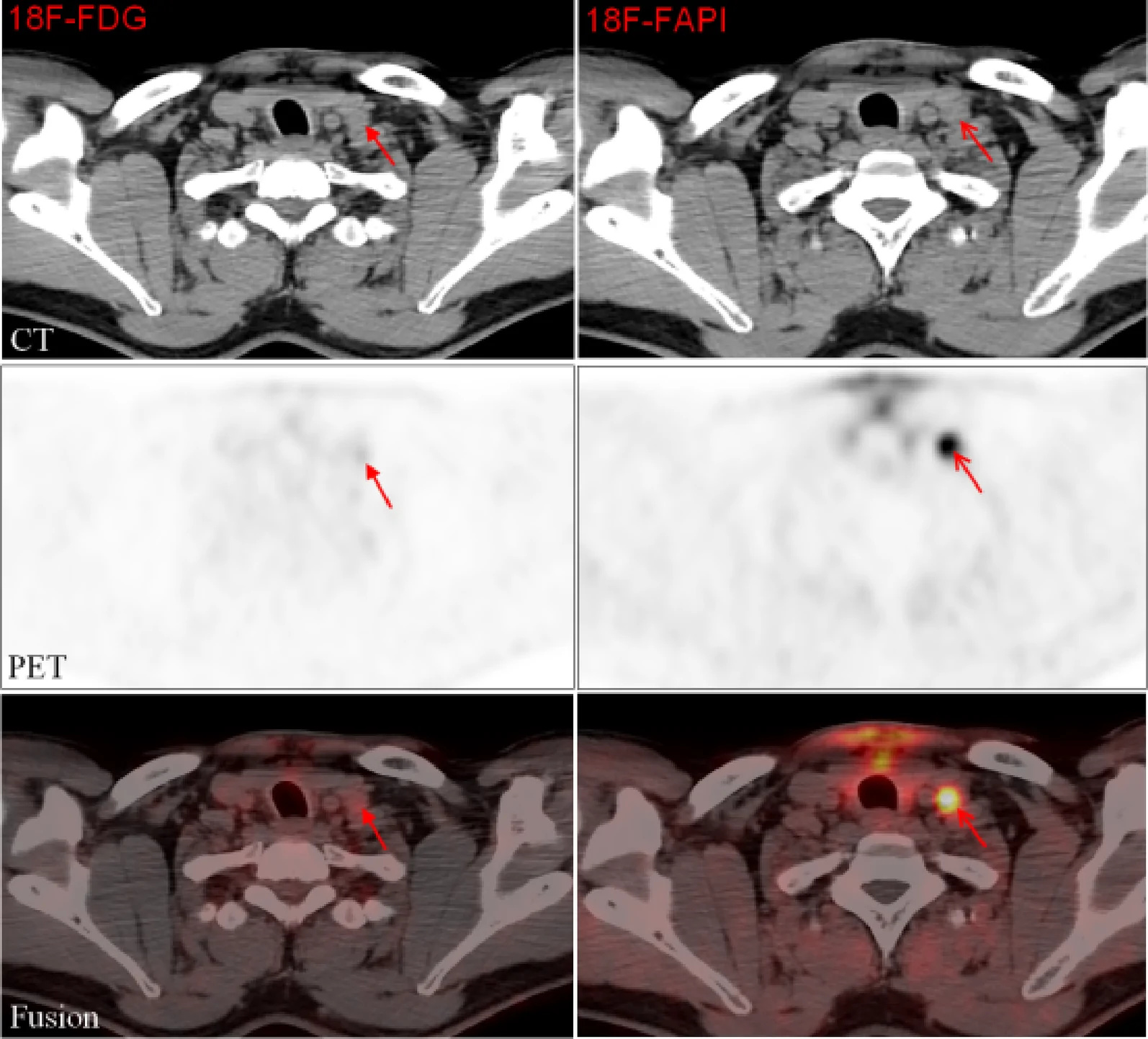
[^18^F] FAPI-46 PET/CT of a 46-year-old male patient who presented with papillary thyroid carcinoma one month after surgery. An enlarged lymph node measuring 15 × 13 mm was identified adjacent to the left carotid sheath. This lesion showed intense [^18^F] FAPI-46 uptake (SUVmax = 12.1, SUVpeak = 7.5) and only mild [^18^F] FDG uptake (SUVmax = 2.3, SUVpeak = 1.9). Subsequent lymph node needle biopsy confirmed papillary thyroid carcinoma metastasis

A total of 23 suspected metastatic lymph nodes were detected in 10 patients. Among them, 6 lesions were confirmed as malignant by postoperative histopathology, and the remaining 17 lesions were verified via 6–12 months standardized imaging and clinical follow-up. Finally, 17 lesions were confirmed as malignant metastatic lymph nodes, and 6 were benign reactive hyperplasia lesions (Fig. 1). Details are summarized in Table 2.

**Fig. 2:**
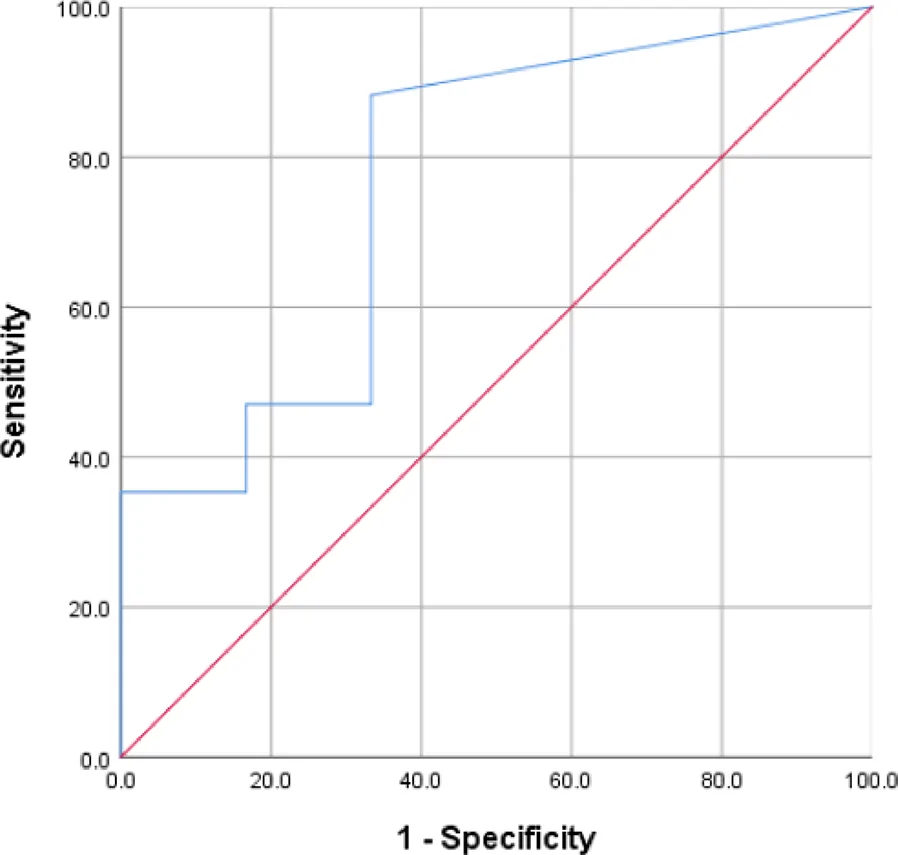
ROC analysis of SUVmax of [^18^F] FAPI-46

For lymph node metastasis detection, [^18^F] FAPI-46 PET/CT achieved a sensitivity of 82.3%, specificity of 66.7% and accuracy of 78.3%, while [^18^F] FDG PET/CT had a sensitivity of 29.4%, specificity of 33.3% and accuracy of 30.4%. McNamar’s test confirmed that [^18^F] FAPI-46 had significantly better diagnostic performance (exact two-tailed *P* = 0.022). For true-positive lymph nodes, [^18^F] FAPI-46 showed significantly higher SUVmax (4.2 ± 3.2 vs. 1.6 ± 2.6, *P* = 0.015), SUVpeak (3.0 ± 2.1 vs. 1.0 ± 1.7, *P* = 0.005) than [^18^F] FDG. ROC curve analysis showed that the optimal SUVmax cutoff value of [^18^F] FAPI-46 for diagnosing lymph node metastasis was 2.3 (AUC = 0.765, sensitivity 88.2%, specificity 66.7%, 95%CI: 0.530–0.999). (Fig. 2)

**Table 2:** Details of 23 suspected metastatic lymph nodes

Lesion No.	[^18^F]FDG SUVmax	[^18^F]FDG SUVpeak	[^18^F]FAP SUVmax	[^18^F]FAPI SUVpeak	Lesion Long-axis Diameter (mm)	Reference Standard (RSTA)	Confirmation Method
1	NA	NA	NA	NA	8	+	Imaging follow-up
2	3.6	2.4	NA	NA	10	+	Imaging follow-up
3	18.4	8.6	NA	NA	9	-	Biopsy pathology
4	8.1	3.9	NA	NA	5	-	Imaging follow-up
5	NA	NA	2.4	2.0	6	+	Imaging follow-up
6	NA	NA	2.5	2.1	6	+	Imaging follow-up
7	2.5	1.9	5.9	5.0	10	+	Biopsy pathology
8	2.5	1.9	6.7	4.3	16	+	Biopsy pathology
9	NA	NA	2.3	2.0	5	+	Imaging follow-up
10	NA	NA	2.3	2.0	6	+	Imaging follow-up
11	NA	NA	2.3	2.0	5	+	Imaging follow-up
12	NA	NA	2.2	1.9	7	+	Imaging follow-up
13	7.6	4.7	2.7	2.2	8	+	Imaging follow-up
14	8.2	5.1	3.9	2.6	10	+	Imaging follow-up
15	2.3	1.9	12.1	7.5	15	+	Biopsy pathology
16	NA	NA	7.4	5.2	15	+	Imaging follow-up
17	NA	NA	5.2	3.3	11	+	Imaging follow-up
18	NA	NA	4.0	2.6	11	+	Imaging follow-up
19	NA	NA	9.2	6.4	10	+	Imaging follow-up
20	NA	NA	3.1	2.5	12	-	Imaging follow-up
21	6.0	3.4	NA	NA	12	-	Imaging follow-up
22	NA	NA	4.5	3.5	11	-	Biopsy pathology
23	6.9	4.2	NA	NA	9	-	Imaging follow-up

RSTA (+) = malignant metastatic lesion; RSTA (-) = benign lesion; NA = not applicable (lesion uptake lower than mediastinal blood pool background)

### False-positive lesions analysis

**Fig. 3:**
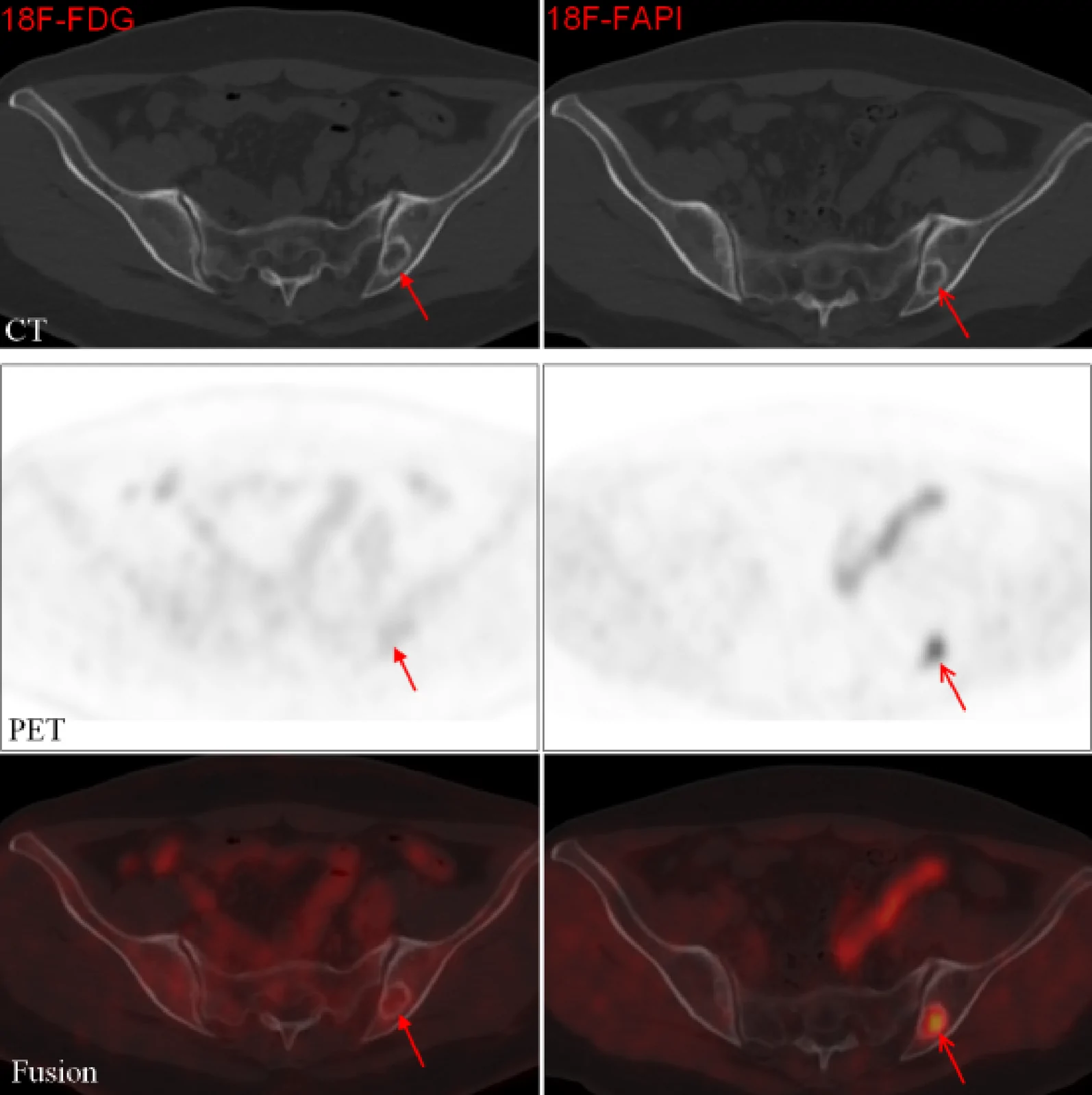
[^18^F] FAPI-46 PET/CT of a 44-year-old female who presented 1 month after total thyroidectomy. The [^18^F] FAPI-46 PET/CT showed a hypodense nodule with a sclerotic border in left ilium. This lesion showed an SUVmax of 6.6 on [¹⁸F]FAPI-46 PET/CT, while no abnormal uptake was observed on [^18^F] FDG PET/CT. Based on the CT features and the follow-up results, it was determined to be a benign bone lesion

No true bone metastasis was detected in all 18 patients. Three false-positive bone lesions with high [^18^F] FAPI-46 uptake (SUVmax: 7.7–7.9, SUVpeak:4.9–5.2) were found in 2 patients, which showed annular sclerotic hyperdense shadows on CT, consistent with benign bone fibroproliferative lesions, and no abnormal [^18^F] FDG uptake was observed in these lesions (Fig. 3). During follow-up, these bone lesions remained stable without malignant progression, confirming benign properties.

[^18^F] FDG PET/CT showed 4 false-positive lymph nodes in 3 patients, with an average SUVmax of 10.1. Among them, 2 lesions showed mild [^18^F] FAPI-46 uptake (average SUVmax = 4.5) and were confirmed as reactive lymph node hyperplasia by puncture biopsy, indicating that [^18^F] FAPI-46 can effectively identify FDG false-positive inflammatory lesions.

### Diagnostic value of sTg for postoperative metastasis

**Fig. 4:**
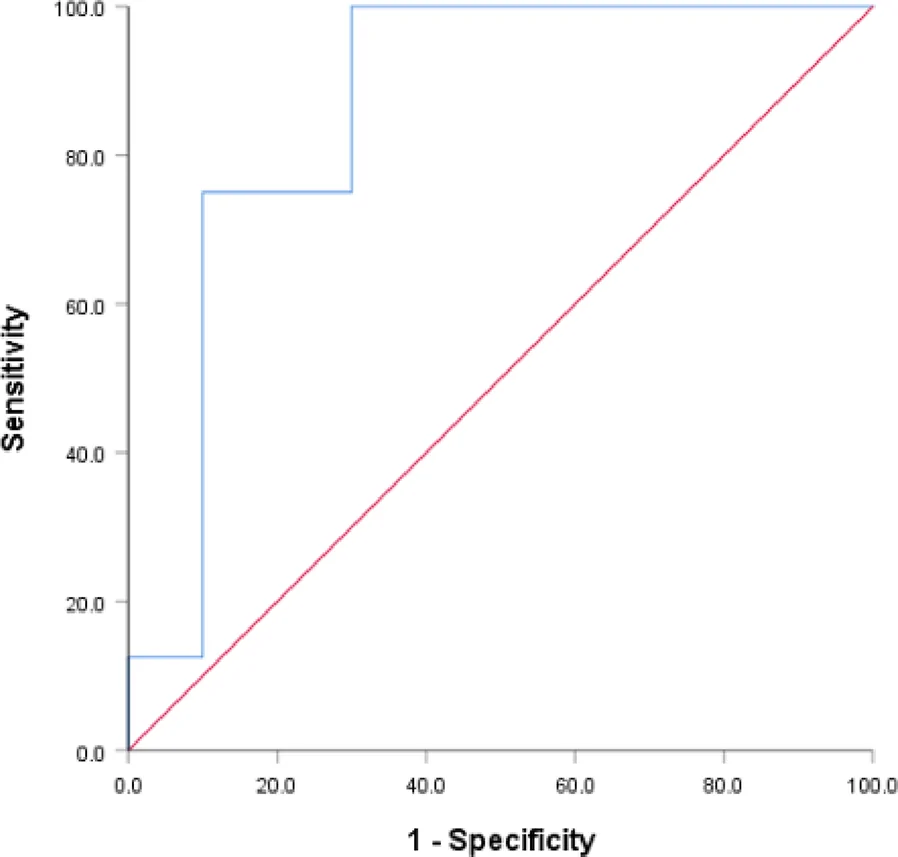
ROC analysis of stimulated Tg level

The sTg level of the metastatic group was significantly higher than that of the non-metastatic group (79.1 ± 51.1 ng/mL vs. 33.9 ± 36.5 ng/mL, *P* = 0.044). ROC curve analysis showed that the optimal sTg diagnostic cutoff value was 25.9 ng/mL (AUC = 0.862, Youden index = 0.7, 95%CI: 0.679-1.000) (Fig. 4). However, this cutoff value was derived from a small single-center sample (*n* = 18), which may have overfitting risk and lacks external validation. All patients with positive [^18^F]FAPI-46 imaging results had sTg > 25.9 ng/mL, suggesting that sTg level has potential guiding value for screening patients suitable for [^18^F] FAPI-46 PET/CT examination, but it cannot be directly used as a standardized clinical diagnostic threshold temporarily. All patients in this study had negative TgAb, eliminating the interference of antibody on sTg detection accuracy.

## Discussion

This prospective head-to-head pilot study focused on postoperative PTC patients before adjuvant ¹³¹I ablation, systematically comparing the diagnostic performance of [^18^F] FAPI-46 and [^18^F] FDG PET/CT in detecting residual and lymph node metastatic lesions. The results fully verify that [^18^F] FAPI-46 PET/CT has significantly superior sensitivity and accuracy for lymph node metastasis detection compared with traditional [^18^F] FDG PET/CT, which is consistent with the unique biological characteristics of FAP-targeted imaging. Different from [^18^F] FDG relying on tumor glucose metabolism (prone to low uptake in well-differentiated PTC and non-specific uptake in inflammatory tissues), [^18^F] FAPI-46 targets CAFs in tumor stroma, which are highly specifically enriched in malignant lesion microenvironment, enabling accurate detection of small-volume residual and metastatic lesions embedded in postoperative fibrotic tissues [9, 10].

In this study, true-positive metastatic lymph nodes showed significantly higher SUVmax and SUVpeak on [^18^F] FAPI-46 imaging than [^18^F] FDG, with better lesion-to-background contrast, which improves the confidence of lesion localization. These finding are consistent with previous studies using ^68^Ga-labeled FAPI tracers in head and neck cancers [11, 12]. The optimal SUVmax cutoff value of 2.3 for [^18^F] FAPI-46 in diagnosing lymph node metastasis provides a preliminary quantitative interpretation basis for clinical application, while large-sample multicenter validation is still needed.

The false-positive results of the two tracers show complementary clinical characteristics. [^18^F] FDG has more false-positive lymph node lesions caused by postoperative inflammation and reactive hyperplasia, while [^18^F] FAPI-46 has fewer inflammatory false positives, showing better diagnostic specificity for malignant lymph nodes. Notably, [^18^F] FAPI-46 has unique false-positive risks in bone lesions: benign bone fibroproliferative lesions can cause high FAP uptake, which cannot be identified by SUV value alone. Therefore, clinical interpretation must combine CT morphological features (annular sclerosis, stable lesion size) for comprehensive judgment to avoid misdiagnosis.

As an important serological indicator, sTg level has good predictive value for postoperative lymph node metastasis [13, 14]. The 25.9 ng/mL cutoff value obtained in this study has good diagnostic efficacy, but limited by the small sample size, this result is only preliminary exploratory data and cannot be promoted as a clinical standard threshold. Meanwhile, we strictly excluded TgAb interference by enrolling only TgAb-negative patients, ensuring the reliability of sTg detection results, which makes up for the deficiency of incomplete serological parameter reporting in previous similar studies. Combined with the 2025 ATA guidelines, sTg can be used as an effective screening tool to select high-risk patients for [¹⁸F] FAPI-46 PET/CT examination, optimizing clinical imaging resource allocation.

Regarding imaging protocol, we performed scans at approximately 60 min post-injection, a standard time point for both tracers based on prior pharmacokinetic studies. While emerging data suggest that FAP-targeted tracers can achieve high tumor-to-background contrast as early as 20–30 min post-injection [15, 16], potentially reducing patient time in the scanner. The dual-tracer (e.g., [^68^Ga] FAPI-46 and [^18^F]-FDG) combined scan in a single session, not only improves sensitivity but also avoids the repeated CT radiation exposure associated with separate scans [17, 18]. These protocols were not evaluated in our study. Exploring earlier imaging time points for [^18^F] FAPI-46 represents a valuable direction for future research to further optimize patient convenience and departmental workflow.

### Limitation

This study has several inherent limitations: (1) Single-center, small sample size (*n* = 18) limits the statistical efficacy and generalizability of the conclusions; (2) Only 6 lymph node lesions were pathologically confirmed, and most lesions were verified by long-term follow-up, which has certain limitations compared with full pathological confirmation; (3) The correlation between [^18^F] FAPI-46 uptake intensity and tumor aggressiveness and long-term prognosis was not analyzed.

## Conclusion

In conclusion, this prospective head-to-head pilot study demonstrates that [^18^F] FAPI-46 PET/CT has significantly better diagnostic performance than [^18^F] FDG PET/CT for detecting postoperative lymph node residual and metastatic lesions in PTC patients before ¹³¹I ablation.

## Data Availability

The datasets used and/or analyzed during the current study are available from the corresponding author on reasonable request.

## Abbreviations

FAPI: Fibroblast activation protein inhibitor
CAFs: Cancer-associated fibroblasts
Rx-WBS: Postablation whole-body ¹³¹I scan
DTC: Differentiated thyroid cancer
sTg: Stimulated thyroglobulin
TgAb: Anti-thyroglobulin antibody
ROC: Receiver operator characteristic curve
AUC: Area under the receiver operating curve
SUVmax: Maximal standardized uptake value
SUVpeak: Peak standardized uptake value
